# Immunophenotypic Profiling of Acute Promyelocytic Leukemia: Insights From a Large Cohort

**DOI:** 10.1002/cnr2.70198

**Published:** 2025-04-19

**Authors:** Supattra Kankhaw, Weerapat Owattanapanich, Orathai Promsuwicha, Thanyakan Thong‐ou, Theera Ruchutrakool, Archrob Khuhapinant, Karan Paisooksantivatana, Smith Kungwankiattichai

**Affiliations:** ^1^ Division of Hematology, Department of Medicine Faculty of Medicine Siriraj Hospital, Mahidol University Bangkok Thailand; ^2^ Center of Excellence of Siriraj Adult Acute Myeloid/Lymphoblastic Leukemia Faculty of Medicine Siriraj Hospital, Mahidol University Bangkok Thailand; ^3^ Department of Pathology Faculty of Medicine Ramathibodi Hospital, Mahidol University Bangkok Thailand

**Keywords:** acute promyelocytic leukemia. *PML‐RARA* fusion gene, decision tree, flow cytometry, immunophenotype, predictive equation

## Abstract

**Background:**

Acute promyelocytic leukemia (APL) is a highly aggressive disease that requires early initial therapy. Rapid diagnosis by flow cytometry remains the mainstay of initial diagnosis. However, the complexity of its immunochemistry has led to diagnostic uncertainty.

**Methods and Results:**

We comprehensively reviewed 2124 AML patients, with 170 classified as APL. In the univariate analysis, HLA‐DR, CD34, CD56, CD11b, and CD11c were predominantly positive in the non‐APL group compared to the APL group, while MPO and CD33 were significantly positive in the APL group. In the multivariate analysis, MPO was identified as a significantly higher positive marker in APL patients, while HLA‐DR, CD34, and CD56 predicted non‐APL patients. The typical immunophenotype of APL, including MPO+/HLA‐DR‐/CD34‐ and CD117+, provided a sensitivity of 51.4%, a specificity of 98.0%, a positive predictive value of 65.8%, and a negative predictive value of 96.5%. By utilizing the decision tree methodology, HLA‐DR, MPO, and CD34 emerged as pivotal indicators for APL diagnosis within this model. Notably, HLA‐DR took precedence, followed by MPO and CD34. Ultimately, a predictive equation for APL diagnosis was proposed to simplify the diagnosis of APL by flow cytometry using the positivity of HLA‐DR, MPO, and CD34 as reference points.

**Conclusion:**

This study underscores the role of immunophenotyping as a rapid and complementary tool to molecular diagnostics, aiding in the preliminary identification of probable APL cases and facilitating timely initiation of therapy.

## 1 Introduction

APL with *PML‐RARA* fusion, a distinct subtype of acute myeloid leukemia (AML), is characterized by the presence of the promyelocytic leukemia‐retinoic acid receptor alpha (*PML‐RARA*) fusion gene [1, 2, 3, 4]. A hallmark complication of APL is disseminated intravascular coagulation (DIC), contributing to its high mortality rate at diagnosis [5, 6]. Timely initiation of all‐trans retinoic acid (ATRA) therapy significantly improves patient outcomes, underscoring the critical need for rapid APL diagnosis [7, 8, 9].

The definite diagnosis of APL relies on identifying the *PML‐RARA* fusion gene, commonly achieved through Real Time Reverse Transcription Polymerase Chain Reaction (RT‐PCR) or fluorescence in situ hybridization (FISH). However, these methods are time‐consuming, typically taking 24‐48 hours to yield results [10, 11].

While the morphology of APL, including the presence of abundant granules and faggot cells, is highly characteristic and can facilitate diagnosis in many cases, reliance on morphology alone may not be sufficient in challenging presentations, such as the microgranular variant or cases with atypical features. Flow cytometry is a rapid tool for acute leukemia diagnosis and aids in AML subtype classification, including APL. Many publications report a typical flow cytometric immunophenotype for APL leukemic populations. They frequently have high side scatter (SSC) property. They express CD13, (bright) CD33, CD117, CD64, and (bright) MPO but lack CD34, HLA‐DR, CD11b, and CD11c [10, 12, 13]. Microgranular APL usually presents with an immunophenotype similar to hypergranular APL, with the exception of CD34, which can be expressed in 75% of patients [6]. However, not all APL cases conform to this typical pattern, indicating the potential for certain markers to possess greater diagnostic value, and combinations of marker positivity or negativity may enhance the accuracy of APL diagnosis.

Many studies have attempted to identify groups of markers that exhibit high diagnostic yield for APL and combine the positivity and negativity among these markers to predict the diagnosis of APL [12, 14, 15]. One example of such studies demonstrated that a well‐known pattern of APL, characterized by the positivity of CD117 and the negativity of HLA‐DR and CD34, provides a Positive predictive value (PPV) of only 70.3% with a negative predictive value (NPV) of 65.3%. Interestingly, the negativity of CD11b and CD11c yields a PPV as high as 98.5% and an NPV of 100% [12]. However, CD11c seems to be less utilized in standard acute leukemia panels. Another study indicated that combined HLA‐DR and CD34 negativity predicts t (15;17) with PPV and NPV of 85% and 100%, respectively. Conversely, PPV and NPV for the positivity of single myeloid markers were lower. For example, CD117 positivity predicts APL with a PPV of 33.73% and NPV of 58.73%, while MPO positivity predicts APL with a PPV of 39.23% and NPV of 57.14% [14].

In this study, we utilized a vast dataset spanning 21 years of flow cytometry data from one of the largest comprehensive care institutions in Thailand. Our objective was to characterize the immunophenotype of APL and discern immunophenotypic differences between APL and other subtypes of AML. Subsequently, we developed an APL predictive equation to expedite the identification of these patients.

## 2 Materials and Methods

### 2.1 Study Groups and Data Collection

We conducted a retrospective review of AML cases diagnosed at Siriraj Hospital, Mahidol University, Bangkok, Thailand, from January 2000 to December 2020. All AML cases with available flow cytometric analysis were included in the study. The gold standard for diagnosing APL was the presence of the *PML‐RARA* fusion gene, as per the 5th edition of the World Health Organization (WHO) classification of hematolymphoid tumors [2]. This diagnosis was confirmed using RT‐PCR or FISH methods.

### 2.2 Flow Cytometric Immunophenotypic Analysis

Flow cytometric immunophenotypic analysis was conducted on fresh bone marrow aspirate specimens collected in ethylenediaminetetraacetic acid‐containing tubes and processed within 24 h using standard staining and lyse/wash techniques. Initially, cell surface antigens were stained, followed by cytoplasmic and nuclear staining. Cells were then fixed, permeabilized, and incubated with antibodies.

A comprehensive panel designed for acute leukemia workup was routinely applied at initial diagnosis. This panel included:

- Myeloid lineage markers: MPO (2D1/MPO421‐8B2), CD13 (L138), CD33 (P67‐6), CD117 (104D2); monocytic differentiation: CD64 (10.1); erythroid precursors: CD235a/Glycophorin A (GA‐R2/HIR2); megakaryocytic precursor: CD41a (HIP8).
- T lymphoid markers: CD3 (UCHT1, SK7), CD5 (L17F12), CD7 (4H9/M‐T701).
- B lymphoid markers: CD19 (SJ25C1), CD20 (L27), CD79a (HM47), CD10 (W8E7).
- Immature markers: CD34 (8G12/581), TdT (E17‐1519), HLA‐DR (L243/G46‐6).
- Non‐lineage‐specific markers: CD11b (D12), CD11c (S‐HCL‐3), CD16 (NKP15/3G8), CD56 (NCAM16.2).

Each marker was selected for its diagnostic relevance, such as identifying immature markers, lineage‐specific markers for myeloid, B, and T lymphoid cells, and subclassification of myeloid leukemia.

Positivity was defined using a multiple gating strategy that incorporated internal controls (e.g., other WBC subsets as a reference), unstained controls, isotype controls for intracellular staining, and a 20% positivity cut‐off for borderline cases to ensure reproducibility and minimize intersample variability.

### 2.3 Statistical Analysis

Statistical analyses were conducted using the SPSS 18.0 software package (SPSS Inc., Chicago, IL, USA). The clinical characteristics were presented as median with interquartile range and as mean with standard deviation or percentage. The relationship between positive and negative *PML‐RARA* gene groups was compared using Mann–Whitney's *U*‐test and unpaired *t*‐test.

A *p*‐value of less than 0.05 (two‐tailed) was considered statistically significant for all analyses. Univariate and multivariate predictions of *PML‐RARA* gene positivity were assessed using binary logistic regression analysis and presented as odds ratios (ORs) with 95% confidence intervals (CIs).

The APL predictive score was derived from a multiple logistic regression model, and a nomogram was utilized to estimate the probability of APL positivity. A receiver operating characteristic (ROC) curve analysis was employed to determine the most suitable cutoff point. Sensitivity, specificity, positive predictive value (PPV), negative predictive value (NPV), Likelihood ratio (LR), and accuracy of this cutoff point were calculated [16, 17].

## 3 Results

### 3.1 Baseline Characteristics

A total of 2124 patients diagnosed with AML were retrospectively enrolled in this study. Among them, 170 patients were identified as having APL based on the presence of the *PML‐RARA* fusion gene. Patients without the *PML‐RARA* fusion gene were categorized as non‐APL patients. The mean age at diagnosis for the entire patient cohort was 44.25 and 49.38 years for APL and non‐APL patients, respectively. Patients diagnosed with APL tended to be slightly younger compared to those with non‐APL AML. Gender distribution was relatively balanced between the two groups. APL patients exhibited lower white blood cell (WBC) counts and platelet counts compared to non‐APL patients. The baseline characteristics are shown in Table 1.

**TABLE 1:** Baseline characteristic.

Characteristic	Non‐APL (*N* = 1954)	APL (*N* = 170)	*p*
Mean age (years)	49.38 ± 18.71	44.25 ± 17.27	0.001
Sex(Male/Female)	982/972	83/87	0.72
Mean Hb (g/dl)	7.84 ± 2.66	8.03 ± 2.51	0.382
Mean Hct (%)	23.74 ± 8.57	23.67 ± 8.13	0.918
Median Wbc (x10^9^/L)	24.70 (7.09–78.37)	18.90 (3.88–62.00)	0.003
Median platelet(x10^9^/L)	45.00 (23.0–84.0)	36.00 (19.65–62.80)	0.011

Abbreviations: Hb, hemoglobin; Hct, hematocrit.

### 3.2 Comparisons of Immunophenotype Profile Between APL and Non‐APL Group

The univariate analysis of HLA‐DR, CD34, CD56, CD11b, and CD11c was predominantly positive in the non‐APL group in comparison to the APL group, while MPO and CD33 were significantly positive in the APL group. The immunophenotype profiles are shown in Table 2.

**TABLE 2:** Flow cytometric immunophenotype profile of AML patients.

Marker	Non‐APL group, *N* = 1954	APL group, *N* = 170	*p*	OR(95% CI)
MPO	773/1799 (43.11%)	140/153 (91.5%)	< 0.001	14.294 (8.04–25.43)
CD34	1331/1953 (68.15%)	24/170 (14.12%)	< 0.001	0.077 (0.049–0.120)
CD33	1813/1941 (93.4%)	167/170 (98.23%)	0.012	3.93 (1.237–12.483)
CD13	1845/1940 (95.1%)	160/166 (96.38%)	0.460	1.373 (0.592–3.183)
HLA‐DR	1788/1954 (91.5%)	8/170 (4.7%)	< 0.001	0.003 (0.001–0.008)
CD11c	1606/1819 (88.3%)	66/153 (43.1%)	< 0.001	0.101 (0.071–0.143)
CD117	1329/1684 (78.91%)	93/130 (71.54%)	0.050	0.671 (0.451–1.000)
CD56	740/1917 (38.6%)	50/168 (29.8%)	0.024	0.674 (0.478–0.950)
CD11b	611/1382 (44.2%)	13/89 (14.6%)	< 0.001	0.199 (0.109–0.362)
CD64	918/1664 (55.17%)	73/122 (59.83%)	0.317	1.211 (0.832–1.761)

All markers with significant positive correlation from the univariate analysis, including MPO, CD34, HLA‐DR, CD33, and CD56, were taken into account for the multivariate analysis. However, CD11b and CD11c were excluded due to lower sample sizes in comparison to other markers. In the multivariate analysis, MPO was identified as a significantly higher positive marker in APL patients, while HLA‐DR, CD34, and CD56 were the ones that predicted non‐APL patients. The results of the multivariate analysis are presented in Table 3.

**TABLE 3:** Univariate and multivariate logistic regression.

Factor	Univariate	*p*	Multivariate	*p*
	OR (95% CI)		OR (95% CI)	
MPO	14.294 (8.036–25.425)	< 0.001	9.83 (5–19.328)	< 0.001
CD34	0.077 (0.049–0.120)	< 0.001	0.515 (0.28–0.947)	0.033
HLA‐DR	0.003 (0.001–0.008)	< 0.001	0.006 (0.002–0.014)	< 0.001
CD33	3.93 (1.237–12.483)	0.012	1.716 (0.021–14.019)	0.621
CD56	0.674 (0.478–0.950)	0.024	0.453 (0.271–7.57)	0.003

### 3.3 Development of Predictive Equation for APL

The predictive equation for APL was developed and assessed through multiple logistic regression models. The selected model provides high sensitivity for diagnosis while maintaining good specificity and using common markers present in acute leukemia across different laboratories. The model is shown below:

- APL score = 2 + (−2*MPO) + (5*HLADR) + (1*CD34).
- Where MPO, HLA‐DR, CD34, CD33 and CD56 = 1 (if positive).
- MPO, HLA‐DR, CD34, CD33 and CD56 = 0 (if negative).
- Score ≤ 4 predict APL with a sensitivity of 96.1%, specificity of 91.3%, PPV of 48.4%, NPV of 99.6%, positive LR of 11.00, negative LR of 0.04, and accuracy of 91.64%.

**FIGURE 1:**
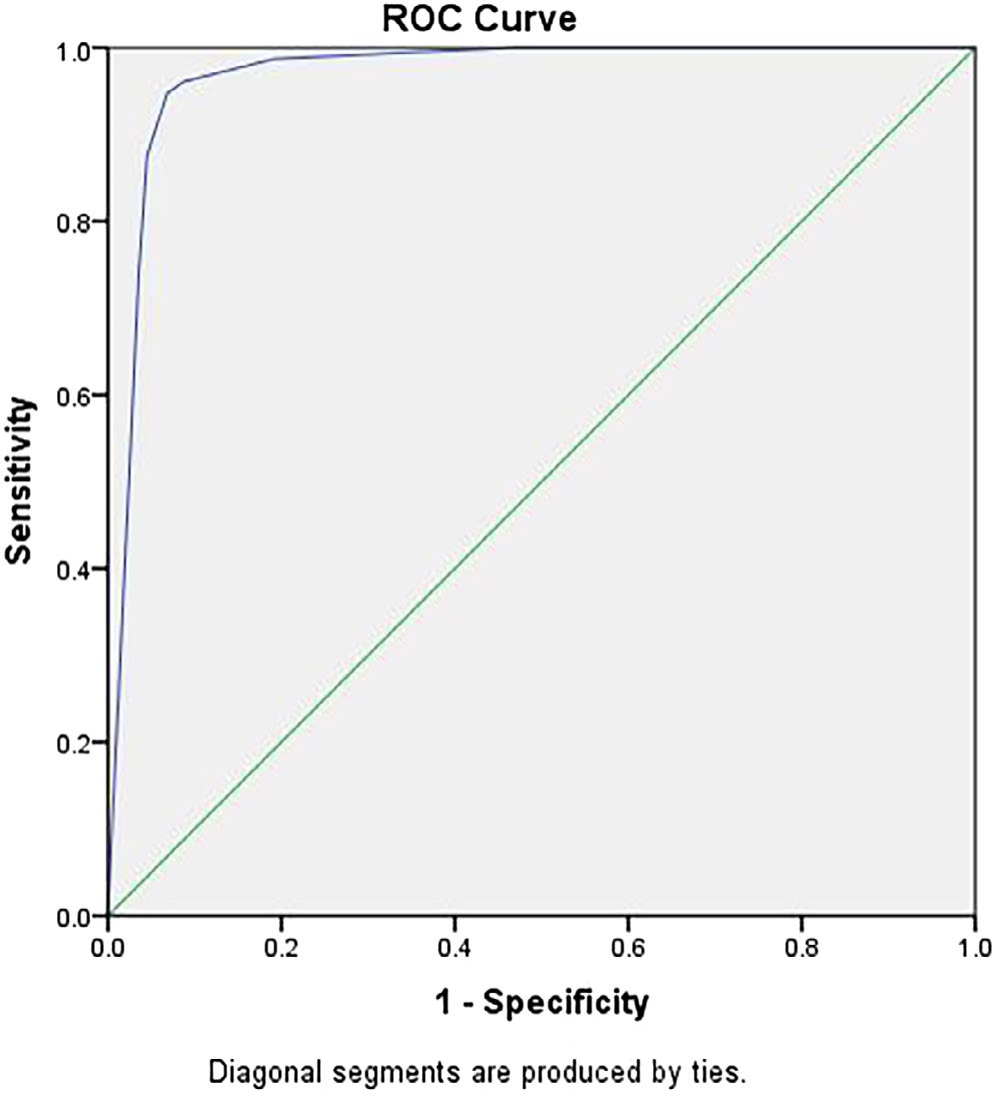
Receiver operating characteristic (ROC) curve analysis of the PML‐RARA gene predictive equation. The area under the curve (AUC) was 0.968 (95% CI: 0.959–0.977), indicating excellent discriminatory ability of the predictive equation for detecting PML‐RARA gene positivity. This ROC analysis was used to determine the optimal cutoff value for classifying positive versus negative PML‐RARA gene status, balancing the sensitivity and specificity of the predictive model.

We use the receiver operating characteristic (ROC) curve analysis of the *PML‐RARA* gene predictive equation. The most suitable cutoff level for *PML‐RARA* gene positivity was obtained when the area under the curve was 0.968 (95% CI was 0.959–0.977) (Figure 1).

### 3.4 Development of Decision Tree Analysis for APL

**FIGURE 2:**
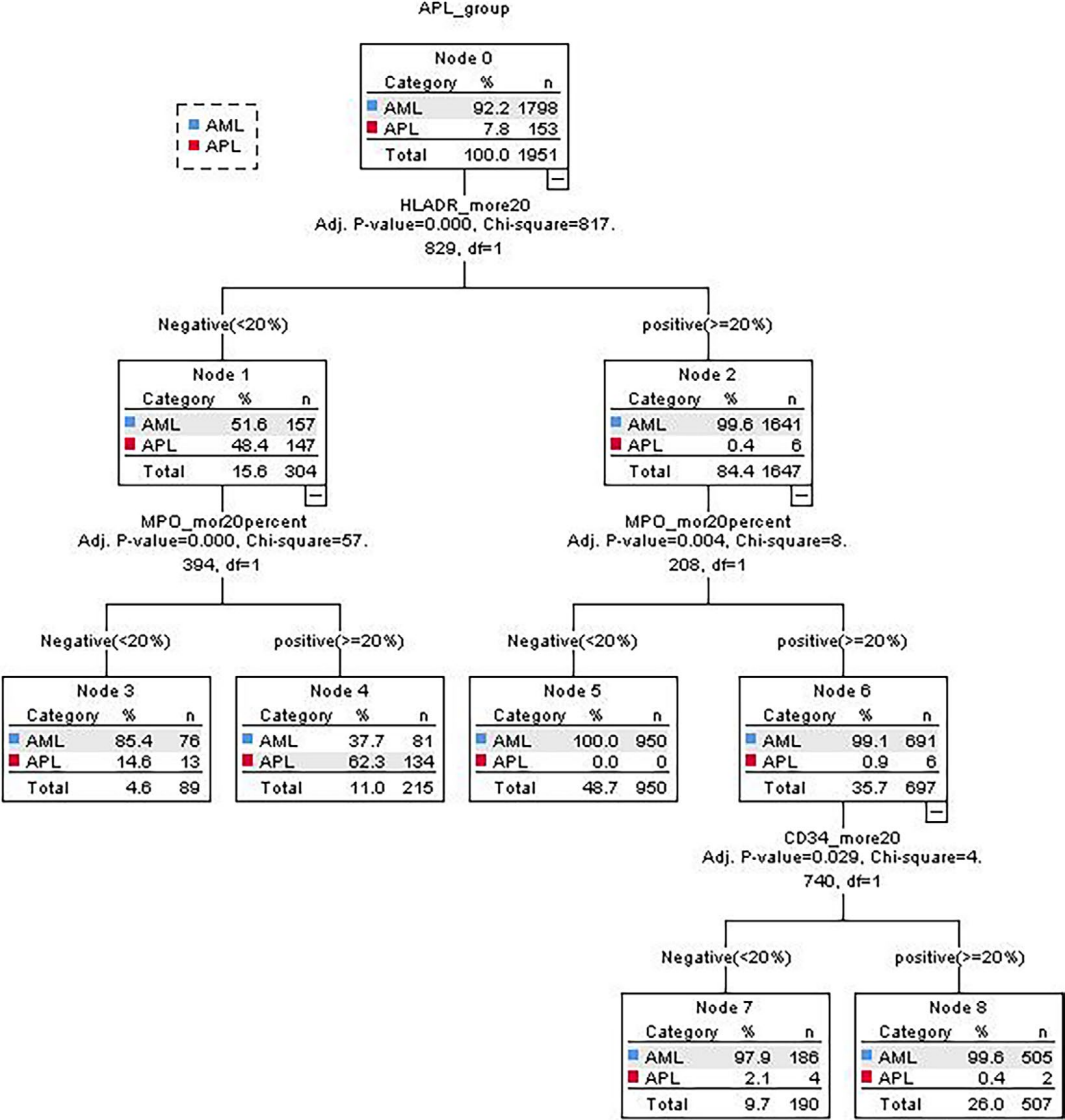
Decision tree model for APL prediction. This hierarchical classification model illustrates the sequential application of three key immunophenotypic markers (HLA‐DR, MPO, and CD34) for APL diagnosis. The model prioritizes HLA‐DR negativity as the primary branch point, followed by MPO positivity, with CD34 as a tertiary determinant in specific branches. This decision algorithm achieved high diagnostic performance with a sensitivity of 87.5%, specificity of 95.49%, positive predictive value of 62.33%, negative predictive value of 98.91%, positive likelihood ratio of 19.44, negative likelihood ratio of 0.13, and overall accuracy of 94.87%. The tree structure visually demonstrates the critical decision paths for clinicians to follow when evaluating potential APL cases based on immunophenotypic flow cytometry results.

The diagnosis of APL was conducted employing a decision tree methodology, yielding a comprehensive model depicted in Figure 2. Among the various markers analyzed, HLA‐DR, MPO, and CD34 emerged as pivotal indicators for APL diagnosis within this model. Notably, HLA‐DR took precedence, followed by MPO and CD34. In cases where HLA‐DR returns negative and MPO positive, the consideration of CD34 becomes less significant. The combination of HLA‐DR negativity and MPO positivity led to the diagnosis of APL with a sensitivity of 87.5%, specificity of 95.49%, PPV of 62.333%, NPV of 98.91%, positive LR of 19.44, negative LR of 0.13, and an overall accuracy of 94.87%.

### 3.5 Investigating the Diagnostic Value of Typical Immunophenotypic Profiles for APL

The typical immunophenotype of APL includes MPO+/HLA‐DR‐/CD34‐ and CD117+. When all of these features are combined, the sensitivity, specificity, PPV, and NPV are shown in Table 4.

**TABLE 4:** Diagnostic value of typical immunophenotypic profiles for APL.

	Non‐APL	APL	Sensitivity	Specificity	PPV	NPV
MPO^+^/HLA‐ DR^−^/CD34^−^/CD117^+^	38/1942 (2%)	73/142 (51.4%)	51.4%	98.0%	65.8	96.5

## 4 Discussion

In this study, we present an analysis utilizing a substantial number of patients from a comprehensive hematology center to evaluate the immunophenotype of APL. Various statistical methods were employed to evaluate the essential markers for rapid APL diagnosis.

The study revealed that the typical immunophenotype of APL, characterized by MPO+, HLADR‐, CD34‐, and CD117+ [3, 8, 12, 18, 19], only achieved a sensitivity of 51.4% for APL diagnosis. This underscores the importance of not relying solely on this typical immunophenotype. However, when present, this immunophenotype demonstrated a specificity of 98% for diagnosing APL.

According to the analysis, HLA DR emerged as the most important marker, followed by MPO. CD34 appeared to be a less important marker compared to these two. One explanation for this could be that CD34 positivity can also occur in APL, particularly in the microgranular variant and bcr3 isoform of *PML‐RARA* [6]. CD117, a marker not statistically significant between APL and non‐APL patients, can be explained by its presence in both myeloblasts and abnormal promyelocytes [13, 19]. Therefore, CD117 should be considered a less important marker for APL diagnosis compared to HLA DR and MPO.

Due to the complexity of immunophenotype evaluation, a predictive equation utilizing three key markers was developed to assist in rapid APL recognition, particularly for technicians or physicians less familiar with comprehensive flow cytometry interpretation. While the predictive equation demonstrated high PPV, the sensitivity was relatively modest. This highlights the utility of the equation as a screening tool to identify probable APL cases, prompting confirmatory molecular testing to ensure accurate diagnosis.

Exploring markers beyond those typically used in acute leukemia panels is intriguing, considering their potential to distinguish APL from non‐APL patients. CD56, for instance, has been suggested in previous literature as a marker distinguishing APL from the cup‐like AML variant by immunophenotype [20, 21, 22]. However, in this study, CD56 was significantly less positive in APL compared to the non‐APL group, consistent with some previous findings [7, 23]. Therefore, it remains uncertain whether CD56 can reliably distinguish APL from non‐APL patients. Nonetheless, its positive expression in APL promyelocytes has been associated with poor patient outcomes [7, 9, 24, 25], as well as with high white blood cell counts, low albumin levels, and the BCR3 isoform [9].

CD11c and CD11b were significantly less positive in APL compared to non‐APL patients. While some reports suggest CD11c as a marker to distinguish APL from non‐APL [12, 13], CD11b is less frequently mentioned as a key marker for APL diagnosis [8, 12, 26, 27]. However, both markers are less utilized in flow cytometry for acute leukemia.

Although this report provides a comprehensive review of APL using various methods, there are limitations. First, the analysis did not consider the intensity of positivity, whether dim, moderate, or bright positive, despite APL being associated with bright MPO and CD33 [3, 18, 28]. However, objective measurement should be prioritized, considering that positivity intensity can vary among laboratories due to factors such as instruments, antibodies, and compensation techniques. Second, this study did not collect data on typical morphological features, such as the presence of abundant granules, faggot cells, or the characteristic teardrop or flame‐shaped appearance of the blast population on the SSC/CD45 plot. While these features are valuable for diagnosing classical APL and should be assessed in all suspected AML cases whenever possible, they are less reliable for identifying the microgranular variant of APL [6, 19, 29]. Furthermore, the inherent subjectivity in defining these characteristics, particularly in borderline cases, emphasizes the need for more objective phenotypic data derived from flow cytometry, which is the primary focus of this study. Lastly, the control group consisted of non‐APL AML cases, which introduces heterogeneity in immunophenotypic and molecular features. While including non‐AML samples as controls could enhance the robustness of the ROC curve analysis, this was not feasible due to the limitations of our retrospective dataset.

## 5 Conclusion

This study underscores the role of immunophenotyping as a rapid and complementary tool to molecular diagnostics, aiding in the preliminary identification of probable APL cases and facilitating timely initiation of therapy.

## Author Contributions

All authors contributed to conceptualization. S.K.1, W.O., O.P., T.T., and S.K.2 contributed to data curation, investigation, and methodology. S.K.1, W.O., O.P., K.P., and S.K.2 performed data analysis. S.K.1, W.O., O.P., and S.K.2 drafted the manuscript. W.O. and S.K.2 were responsible for project administration and supervision. T.R., K.P., W.O., and S.K.2 reviewed the manuscript and provided comments and conclusions. W.O. and S.K.2 edited the manuscript. W.O. served as the essentially intellectual contributor. S.K.2 served as the corresponding author. All authors have accepted responsibility for the entire content of this manuscript and approved its submission.

## Ethics Statement

This study was approved by the Siriraj Institutional Review Board, Faculty of Medicine Siriraj Hospital, Mahidol University (Certificate of Approval number: si 291/2021). The board waived the requirement for informed consent due to the retrospective nature of the study, minimal risk to subjects, and no adverse effect on subjects' rights and welfare.

## Conflicts of Interest

The authors declare no conflicts of interest.

## Data Availability

Data sharing not applicable to this article as no datasets were generated or analysed during the current study.
